# A dual-mode neurostimulation approach to enhance athletic performance outcome in experienced taekwondo practitioners

**DOI:** 10.1038/s41598-022-26610-3

**Published:** 2023-01-05

**Authors:** Ali-Mohammad Kamali, Mojtaba Ijadi, Behnam Keshtkarhesamabadi, Milad Kazemiha, Reza Mahmoudi, Amrollah Roozbehi, Mohammad Nami

**Affiliations:** 1 Neuroscience Laboratory, NSL (Brain, Cognition and Behavior), Department of Neuroscience, School of Advanced Medical Sciences and Technologies, Shiraz University of Medical Sciences, Shiraz, Iran; 2 Iranian Neuroscience Society-Fars Chapter, DANA Brain Health Institute, Shiraz, Iran; 3 Instituto de Investigaciones Científicas Y Servicios de Alta Tecnología (INDICASAT AIP), City of Knowledge, Neuroscience Center, Panama City, Panama; 4 Cellular and Molecular Research Center, Yasuj University of Medical Sciences, Yasuj, Iran; 5 High Performance Brain, Helena Félix Street, No. 7 to 7 D, 1600-121 Lisbon, Portugal; 6 Harvard Alumni in Healthcare, Harvard University, Boston, MA USA; 7 Brain, Cognition, and Behavior Unit, BrainHub Academy, Dubai, United Arab Emirates

**Keywords:** Neuroscience, Cognitive neuroscience, Motor control

## Abstract

Transcranial Direct Current Stimulation (tDCS) is a growing empirical approach to improve athletic performance. Some recent studies have investigated the effects of transcutaneous spinal direct current stimulation (tsDCS) on the motor performance such as reaction time. TDCS and tsDCS can lead to alteration of the spontaneous neural activity, and the membrane potentials of motor neurons in cerebral cortex and spinal interneurons, respectively. Given the paucity of experimental studies on the non-invasive brain stimulation in the field of sports neuroscience, especially martial sports, the present study aimed at investigating the effects of neurostimulation in potentiating the motor and cognitive functions in experienced taekwondo practitioners. The study sample included 15 experienced male taekwondo players who received real or sham direct current stimulation on the primary motor cortex (M1) and the lumbar spinal segment (T12-L2) over two sessions, 72 h apart. Next, the performance of the participants was evaluated through a simulation of taekwondo exercise directly after the sham and real sessions. Moreover, a cognitive platform (CBS: Cambridge Brain Science) was used to investigate the participants’ cognitive profile in each instance. Unlike sham stimulation, real tDCS was associated with improved selective attention and reaction time in both in the simulated task performance and cognitive examination. The concurrent cortical and trans-spinal tDCS was found to improve selective attention (31% performance improvement) (*P* < 0.0001) [EFFECT SIZE; 1.84]. and reduce reaction time (4.7% performance improvement) (*P* < 0.0001) [EFFECT SIZE; 0.02]. Meanwhile, the intervention failed to leave a significant change in cognitive functions evaluated through CBS (*P* > 0.05). As informed by our results, the present dual-mode neurostimulation could improve motor functions potentially through the effect of tsDCS over the spinal interneurons and tDCS over the primary motor cortex. Likewise, our findings suggested an improved performance in simulated taekwondo task after real- but not sham-stimulation. This study paves the way for designing neurostimulation protocols to improve the performance of professional athletes, namely martial art practitioners, including their accuracy and velocity of reactions. Such positive effects of neuostimulation in athletic performance as demonstrated in this research and similar reports are expected to enhance the athletes’ success in professional competitions.

## Introduction

Transcranial Direct Current Stimulation (tDCS) is a method of brain stimulation that transfers a weak electric current (in the range of some milliamperes) from the scalp to the brain tissue. This electric current is partially transmitted to the brain tissue, which affects neuronal activities. As a non-invasive tool for the stimulation of cerebral neuronal flexibility, tDCS is also used in research on psychiatric and neurologic disorders such as epilepsy, migraine headache, stroke, and depression^1^. Transcutaneous spinal direct current stimulation (tsDCS) is another non-invasive method of stimulation of the central nervous system (CNS), considered as a novel approach to spinal function stimulation or inhibition. The effect of tsDCS on the spinal white matter tracks and the excitability of the spinal motor neurons have been reported lately^2,3^. TDCS studies are mainly focused on patients, and relatively few studies have assessed the effects of tDCS on healthy individuals. Studies have shown that tDCS can effectively improve cerebral functions, such as information processing, memory capacity, verbal and methematical skills, motor activities, creativity, attention, accuracy, and effective learning^4–6^.

Given the the effects of such a type of brain stimulation on healthy individuals, researchers are increasingly interested in amalgamating sports sciences and neurosciences in terms of pursuing non-pharmacological approaches such as brain stimulation to improve athletic performance^7–9^.

With respect to neuromedical research, The effect of tDCS has been well established in the rehabilitation of stroke patients, where the combination of motor training and tDCS becomes significantly effective in achieving improvement in such patients^10^. In addition, studies have shown that transcranial electrical stimulation of the motor cortex improve movement and balance in patients with Parkinson's disease and stroke^11,12^. Meanwhile, achieving functional goals in these patients require complex tasks and improved dynamic balance^13^. With respect to cognitive predicaments, another study on 64 patients with attention deficit/hyperactivity disorder showed that tDCS-treated individuals had significantly improved symptoms of inattention as compared to a sham group^14^. In the same vein, a systematic review highlighted that tDCS along with physical training can effectively increase the excitability of the motor cortex, physical performance and motor learning in healthy individuals^15^. Specifically, the latter report and similar studies in the same domain, lead to the emerging idea of incorporating neurostimulation into the field of sport science.

Martial sports are popular in most countries, attracting a remarkable audience each year. Thus, improving the quality and performance of martial sports athletes is highly important. Taekwondo is among the most popular martial sports. It has changed from a martial art used for self-defense in wars (in Korea) to an international sport^16–20^. Powerful kicking techniques are commonly used in taekwondo^17^. Martial Arts and their corresponding philosophies, aspects, concepts, and training methods are largely influenced by culture, geographical region, and history. Today, the top five countries in the field of martial arts are China, Japan, India, Korea and Brazil^21^

Accuracy, agility, and endurance are highly important in taekwondo. In other words, reaction time, attention, and visual-spatial memory of the athletes are expected to significantly affect the results of the taekwondo competitions. That said, and to our best knowledge, the effects of tDCS and tsDCS on taekwondo practitioners have not been investigated yet. Therefore, the present study aimed to assess the effect of dual-mode neurostimulation (concurrent tDCS and tsDCS) on the motor and cognitive function of experienced taekwondo practitioners.

## Methods

### Study participants

The present study was conducted on experienced male taekwondo practitioners with a minimum of 2 years of constant training, at least 3 sessions a week. Sampling was made using clustered randomized sampling, and 15 experienced taekwondo practitioners were randomly selected in Shiraz. The present study was a single-arm double-blinded trial, and the performance of each participant was randomly evaluated in two real and sham stimulation sessions. (Table 1). with respect to sample size calculation and enrollment, we referred to the earlier related reports^7,22,23^ in which the sample size ranged from 8 To 16 The Kelsey and Fleiss sample size calculation formula^24^ was used (power 80 and α = 0.05) whereby the minimum justifiable number of 14 participants were decided to get enrolled. Based on earlier reports in the field of sport, the specialized population limited the sample size of the study.

**Table 1:** Participants’ demographic information (n = 14), Mean ± SEM (standard error of mean).

Mean age in years	23.5 ± 3
Mean years of training in Taekwondo	5.2 ± 2.1
Mean weight (kg)	67.4 ± 19
Mean height (cm)	180 ± 7

### Inclusion criteria

The study participants did not reveal any history of psychiatric diseases and had not received medication that could affect cognitive or motor functioning in the past 3 months. They were contacted a day before the study to remind them to have quality sleep and observe sleep hygiene measures. They were briefed to maintain their routine diet on the experiment day. The study process was explained to the participants, and they signed an informed written consent based on the Helsinki declaration based on which the athletes were allowed to quit the study at any point. The approval of the research ethics committee was obtained from Yasuj University of Medical Sciences with the research ethics code of IR.YUMS.REC.1400,176 and the entire study was conducted in compliance with the Standards for Ethics in Sport and Exercise Science Research laid down by Harriss et al. in 2019^25^.

### Study design

**Figure 1:**
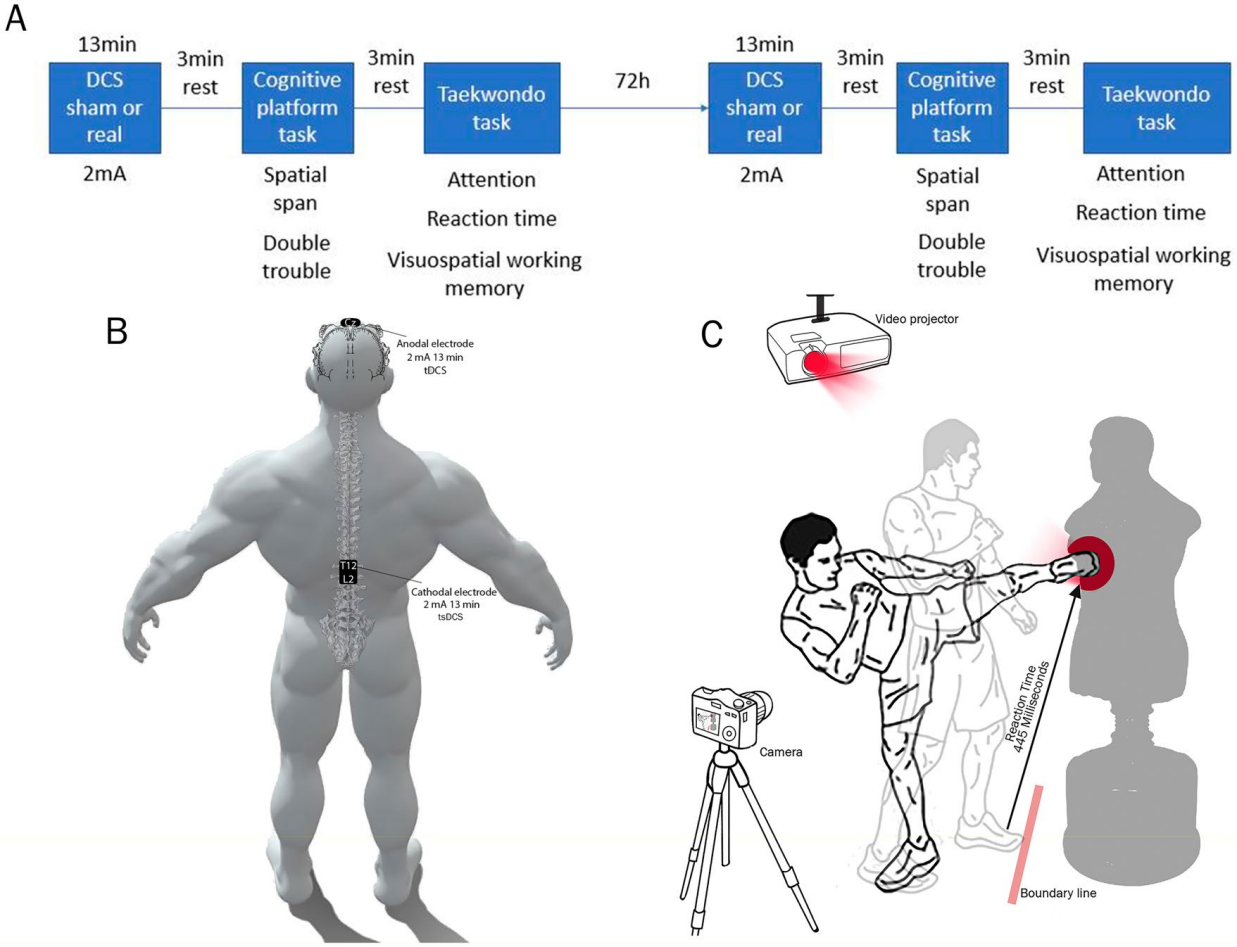
Study protocol, the tDCS + tsDCS montages used for brain stimulation and Taekwondo task. (**A**) Design of the steps and order of the test for study participants. A 3-min rest was considered between the steps. (**B**) Electrodes placement. Anode electrode was placed on the scalp on the primary motor cortex of the lower limb (M1), and the cathode electrode was placed on the skin of the twelfth thoracic to second lumbar vertebrae (T12-L2) on the lumbosacral plexus. (**C**) Design of the motor tests of the participants. The distance between the athletes and the mannequin and its height was adjusted based on the height of the athlete. The motor tests and motor stimuli were shown on the mannequin by the video projector, and reaction time, accuracy, and visual-spatial memory were assessed accordingly. All activities of the athletes during the tests were recorded by a video camera at 240 frames per minute.

**Figure 2:**
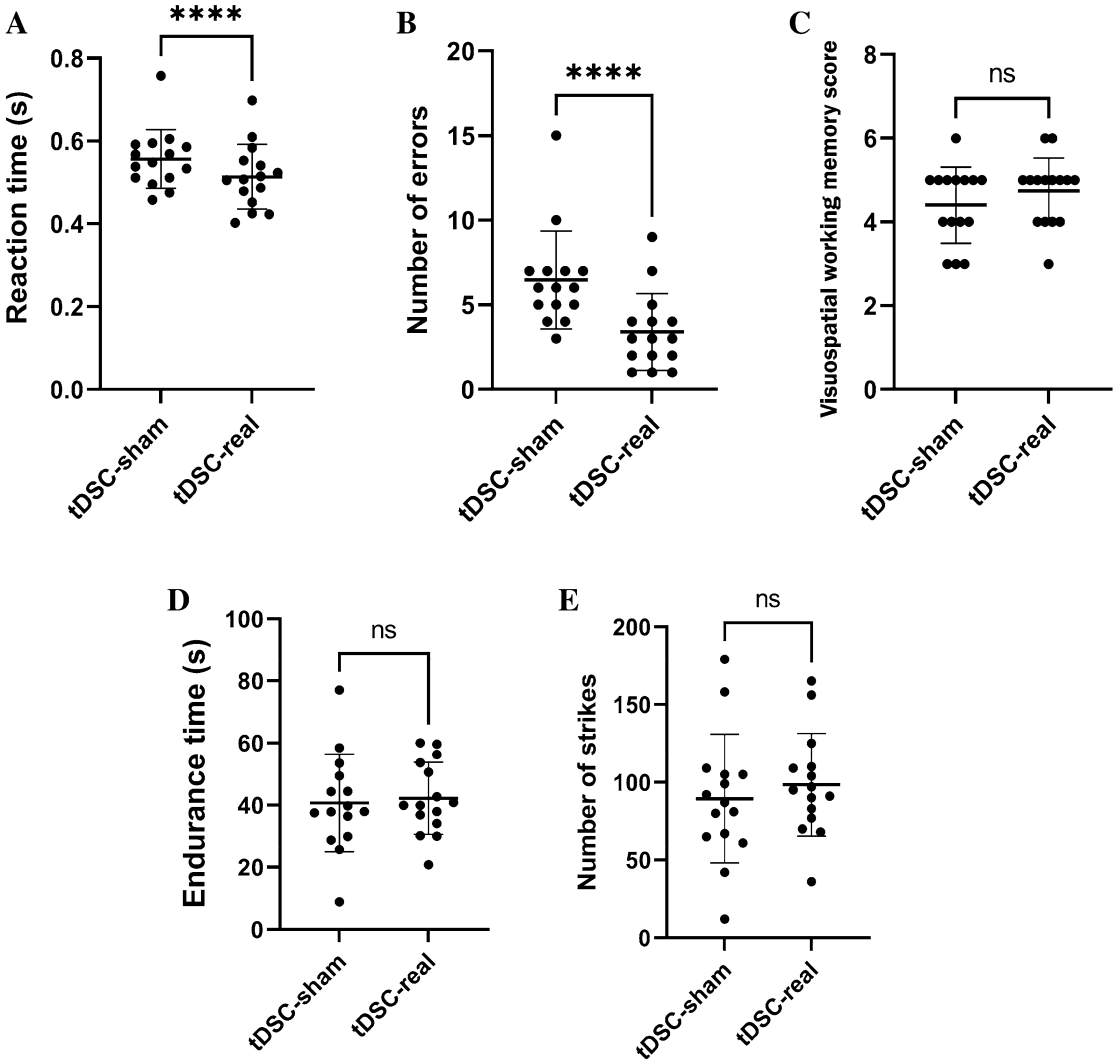
(**A**) Dot plots representing the participants’ performance for the reaction time task. (**B**, **C**) Dot plots representing the participants’ performance for the selective attention and visuospatial memory tasks. (**D**) Endurance time: Athletes had to successively hit the mannequin, and the test was finished when the athlete put the dominant leg on the ground. (**E**) The number of strokes of the athlete during the endurance task. ****Significant; (*p* < 0.0001) ns: Non-significant.

The present double-blind trial was conducted over the course of two sessions. Participants and researchers were blinded regarding the intervention plan of the sessions. The participants were submitted to cognitive tests and performance tasks both after the sham- and real-tDCS sessions. The sequence through which each participant received either sham- or real-tDCS remained randomized and blinded. Data were collected over 2 sessions, 72 h apart. To mitigate the effect on participants' learning, those who received sham stimulation in the first session received tDCS + tsDCS in the second session and vice versa. After cerebral stimulation, the taekwondo practitioners were asked to perform 2 cognitive tasks of the Cambridge Brain Sciences- Cognitive Platform (CBS-CP). The hemodynamic response of the frontopolar region (FP1 region according to the International 10–20 system of EEG). Next, participants exercised for 10 min (including stretching and jumping) and were briefed about the stimulation motor tasks (Fig. 1A). Finally, after a 3-min rest, taekwondo practitioners were submitted to 4 motor tasks, including selective attention (The ability to attend to a specific stimulus or activity in the presence of other distracting stimuli)^26^, reaction time (Defined as the time between a stimulus and a response)^27^, visual-spatial memory (The component of working memory that allows you to temporarily hold and manipulate information about places)^28^, and resistance. Figure 2 depicts the design of the motor test for the participants.

### Dual neurostimulation (tDCS + tsDCS)

The sham or real value of tDCS + tsDCS was applied to the brain and spinal cord by an electrical stimulation device (Segal Stim Farmed Tajhiz 2-channels, Output current accuracy: ± 5μA, Power supply: 3.7 Li-ion rechargeable battery, Maximum voltage: 28 V). In each session, anode and cathode electrodes were connected to the target sites using moist sponge pads. The connection sites were cleaned with alcohol. Th anode electrode (8.5 × 5.5 cm) was placed on the scalp on the primary motor cortex of lower limb M1 (CZ according to the International 10–20 system of EEG), and the cathode electrode (8.5 × 5.5 cm) was placed on the skin overlaying the twelfth thoracic to second lumbar vertebrae (T12-L2) on the lumbosacral plexus (Fig. 1B). In the real stimulation session, a 2 mA current was applied for 13 min. In the sham stimulation session, under the same setting, the current was applied only for 30 s to induce the sense of stimulation, though the device was on for 13 min.

The duration of treatment sessions using tDCS is among the key variables defining the modulatory effects. Nitsche et al. showed that the effects of a tDCS session (2 mA, 13 min) continued to remain for 150 min^29^. In addition, a study on cyclists and one of our earlier research works on bodybuilders and boxers indicated the effectiveness of 13 min of stimulation in enhancing athletic performance^8,22,30^. As such, we considered ‘13 min’ as an optimal stimulation duration already examined. It is worth noting that the length of stimulation was 20 min in most studies. We considered a shorter length of stimulation as it could be more convenient before sport competitions.

### Cognitive examination

A cognitive examination was done immediately after brain stimulation. To do so, two tests, including short-term memory (Spatial Span), and selective attention (Double Trouble) tests, were selected from the online Cambridge Brain Sciences- Cognitive Platform (CBS-CP) (www.cambridgebrainsciences.com), reflecting the effect of the brain stimulation protocol on our cognitive domains of interest (i.e. working memory and selective attention).

### Taekwondo motor examination (Selective attention, reaction time, short-term memory)

Throughout the motor tests, colored circles and luminous circles (red, blue, yellow) were shown on the mannequin using a video projector. In the reaction time test, the athlete stood before the line, though in the rest of the motor tests, including concentration, memory, and resistance time, the distance between the athletes and the kick point on the mannequin was adjusted based on their comfort. Moreover, the height of the mannequin was adjusted based on the height of the athlete (Fig. 1C).

#### Selective attention

This task was designed to evaluate the selective attention of athletes following real or sham stimulation. Participants were asked to do exercises first, and they were trained on how to do the test. In this test, three blinking circles (red, blue, and yellow) were shown on the mannequin in front of the athletes for 3 min. Before the test, the dominant leg of the participants was determined by self-report, i.e., preferred use of one leg over the other. The athletes were asked to hit the red circles with their dominant leg and the blue circles with their non-dominant leg. Athletes should not hit the yellow circles, and hitting yellow circles was considered an error. Colors were randomly shown on the mannequin, and the interval of showing colors was unpredictable. The test lasted 180 s, and the whole process was recorded by a sports video recorder (1080p 240 frames per second). After recording the performance of the athletes, the number of correct and wrong hits was counted for statistical analysis.

#### Reaction time

This test is designed to assess the velocity of the dominant leg. The dominant leg was self-reported as the leg with higher skill and precision for taekwondo techniques since it has been proved that this definition has the highest effect in determining the differences between limbs in controlling the states of a leg or hand^31^. Then, the participants were asked to stand before the predetermined line (the distance to the mannequin), which was similarly done in both real and sham sessions. The distance was calculated based on the weight and height of the athlete. Then, 10 colored luminous circles were shown at random intervals on the mannequin for 75 s. The interval between the emergence of the circle and the hitting of the circles is the reaction time index. The limb movement of the athletes was manually tracked using Kinovea software (version 0.8.15) at 240 frames per second. The mean reaction time for each of the 10 kicks was used for the final analysis.

#### Short-term memory

At any stage of this test, 4 to 9 colored luminous circles appeared on the mannequin in ascending order. Participants should remember the sequence of circles' appearance, then hit the appearing circles using the dominant leg. The longest correct hits were recorded during the test for statistical analysis.

#### Endurance time

This test was used to investigate the muscular resistance of the lower limbs of the athletes. Participants were asked to hit the mannequin with their dominant leg, which was determined in the reaction time test, while trying to maintain their balance with the supporting leg. Athletes had to successively hit the mannequin, and the test was finished when the athlete put the dominant leg on the ground. Then, the total duration of the test, the number of kicks, and the perceived exertion were measured using the Borg RPE Scale. Note that during the three tests of attention, reaction time, and resistance time, motivating words (e.g., "perfect," "good," "great") were used by the examiner.

### Statistical analysis

The datasets were evaluated for the normality of distribution and homogeneity of variance including the indicators of kurtosis, skewness and Kolmogoprov-smirnov. Accordingly, parametric tests e.g. parried t-test were applied and their equivalent non-parametric tests (e.g. Wilcoxon test) were used in case of non-normal datasets using SPSS version 22.

## Results

In this study, 15 volunteer professional taekwondo players were investigated and underwent the performance and cognitive tests in two days, 72 h apart.

### Reaction time

A significant difference was found between the real and artificial DCS sessions in terms of reaction time (RT) indicating 4.7% performance improvement in terms of reaction time to visual stimulus (Fig. 2A).

### Selective attention

A significant difference was found between the real and artificial DCS sessions in terms of selective attention demonstrating 31% performance improvement in terms of reduced errors in the selective attention test (Fig. 2B).

### Visual-spatial working memory

No significant differences were observed in terms of visual-spatial working memory between the first and second days of real and artificial DCS sessions (*p* > 0.05). (Fig. 2C).

### Endurance time

No significant differences were observed in terms of endurance time between the first and second days of real and artificial DCS sessions (*p* > 0.05) (Fig. 2D).

### Number of kicks during the endurance time

No significant differences were observed in terms of number of kicks during the resistance time between the real and artificial DCS sessions (*p* > 0.05) (Fig. 2E).

### Cognitive evaluation

**Figure 3:**
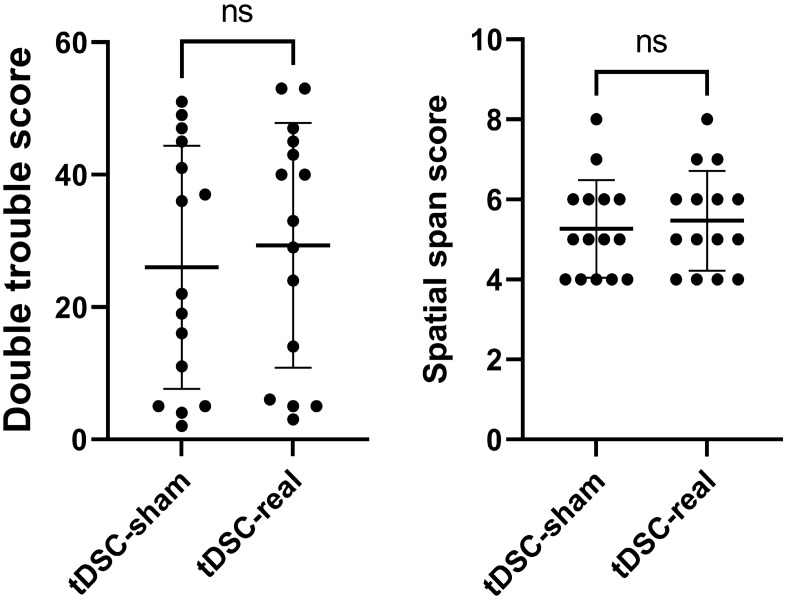
Dot plots representing the participants’ performance for the cognitive behavioral assessment.

Results of the present study showed no statistically significant differences between real and artificial DCS session in terms of Spatial span and Double trouble (*P* < 0.05) (Fig. 3).

Tables 2 summarizes mean and standard errors of mean, as well as the effect size for the variables upon cognitive evaluation and task performance results in sham and real tDCS groups.

**Table 2:** Sham- vs. Real-tDCS effect sized and significance on cognitive performance and task related scores among participants.

Cognitive domain	Sham-tDCS performance-score (Mean ± SEM)	Real-tDCS performance score (Mean ± SEM)	Effect size	Significance
Reaction time	0.55 ± 0.17	0.51 ± 0.18	0.02	*P* < *0.0001*
Selective attention	6.47 ± 6.1	3.4 ± 5.2	1.84	*P* < *0.0001*
Visual-Spatial working memory	4.4 ± 1.5	4.73 ± 1.4	− 0.37	*P* < *0.17*
**Task performance**				
Endurance time	40.6 ± 35.1	42.2 ± 27.5	− 0.22	*P* < *0.55*
Number of kicks during the endurance time	89.4 ± 79.4	98.4 ± 63.6	− 0.53	*P* < *0.06*

## Discussion

The principles of "Faster, Higher, Stronger" are the cornerstones of most competitive sports. In recent years, researchers have been interested in the association of sports sciences and neurosciences using neuromodulation. Neuroplasticity is a key driver in gaining new functional skillsets in amateur and professional athletes. In that sense, evidence have supported the notion that pursuing neuromodulation using technologies such as tDCS and tsDCS might be attributed to an altered spontaneous neural activity and membrane potentials of the cortical and corticomotoneuronal cells, respectively^3,32^. The present study is the first study on dual-mode neurotransmission (tsDCS-tDCS) in martial sports. In the present study, tsDCS-tDCS have been used to assess the hypothesis that this dual-mode neurotransmission can improve the performance of taekwondo practitioners. In the present study on taekwondo practitioners, it was generally shown that anodal tDCS and cathodal tsDCS might help taekwondo practitioners improve their overall performance.

tDCS is a growing approach to improving the performance of healthy individuals. Moreover, recent studies have shown the effects of tsDCS on the motor functions, such as reaction time^33^.

Our other study showed that the stimulation of brain areas could reduce the perceived exertion and improve the strength of the athlete in strength sports^8^. Moreover, our other study showed improved shooting accuracy in professional shooting athletes using tDCS^7^.

In another study, we showed that the stimulation of the spinal cord and primary motor cortex could improve the performance of the upper limb in boxing athletes^30^.

Taekwondo athletes were investigated in the present study since the improved performance of the lower limbs using neurostimulation had not been investigated in martial sports. Also, few studies have applied dual-mode neurostimulation.

The similarity of the study design to real training of the athletes is highly important. Hence, in the current study, it was attempted to simulate the exercises of Taekwondo athletes. Therefore, a special mannequin used by taekwondo practitioners for training was used as well as the visual stimulus. In brief, in this study, the improvement of sports performance in reaction time and selective attention was reported in professional taekwondo practitioners using tsDCS + tDCS.

Reaction time to environmental stimuli is highly important and effective in most sports, especially in martial sports and competitions. Athletes with higher reaction times are one step forward from their competitors^34^.

Resistance is the other important factor. During the fight, the martial athlete should be able to maintain mental and physical readiness. Thus, resistance plays an important role in taekwondo fights^35^. Thus, the effect of dual-mode neurostimulation was investigated on the resistance of the participating athletes.

It can be stated that the most important factor which decides the fight outcome is the accuracy of the kicks, which is improved by long-term training and persistence in implementing the techniques. In addition, increased accuracy of kicks in a fight is associated with higher scores changing the result of the fight in the athlete's favor.

Studies have proved that attention, or the ability to focus on what is happening in the field while ignoring distractions, is a critical factor for perfect performance in sports^26^. For this purpose, the present study was designed to investigate the athlete's accuracy and errors.

Previous studies have demonstrated that tsDCS or tDCS can separately improve the performance of healthy individuals or athletes^2,6,30^. Accordingly, simultaneous tsDCS + tDCS is assumed to have a synergistic effect in enhancing the athlete's performance. However, further studies are required to examine the effect of stimulation of the brain and spinal cord both simultaneously and separately in other groups. In the present study, we could not divide the participants into several groups (brain stimulation, spinal cord stimulation, and simultaneous brain and spinal cord stimulation) since the number of taekwondo practitioners was limited. Thus, the effects of dual-mode neurotransmission (tsDCS + tDCS) were investigated.

While neurologic medications may potentially be used to improve cognitive function^36^, dsDCS and tDCS are the other methods with probably fewer side effects^37^. Although electrical stimulation of the brain was primarily used to help brain-damaged patients^38^; yet, in the past few years, it has also been used in many studies for healthy individuals as well^39,40^. Using this method, different regions of the brain may be stimulated and strengthened using a controlled electric current. It has been demonstrated that brain stimulation is able to enhance the formation of neural synapses and enhance brain functions, including learning, memory, and concentration^41^. Even it has even been reported that brain stimulation may enhance the ability to learn a second language^42^.

In the current study, the performance of athletes in selective attention tests and reaction time were improved by 31% and 4.7%, respectively. Note that improved performance by a few percentages is highly important in professional athletes and may be used in world-class competitions for winning medals.

In 2013, Furuya et al. concluded that the maximum motor learning capacity of professional pianists is limited and may not be improved using the tDCS technique^43^. Despite these results, professional martial practitioners were invited to the present study to determine whether there is a similar capacity limitation in improving their motor performance using neurostimulation. Can they achieve higher levels of sports performance? Moreover, can neurostimulation also disturb cognitive functions?

Since cognitive functions are so vital for athletes, studies had to assess the adverse effects of neurostimulation on the athletes' cognitive performance. Thus, a validated cognitive platform was used in the present study (Cambridgebrainsciences) to recognize the potential positive or negative effects of the brain neurostimulation protocol on the cognitive functions of the participants. This platform consists of three sections: reasoning, memory, and verbal skills. One test was selected from each section to assess the effect of DCS on the cognitive aspects. It was revealed that the suggested brain stimulation dual-mode neurostimulation did not negatively affect the participants' cognitive functions. The effects of a tDCS session persisted for nearly up to 2 h^44,45^. Thus, based on the time limit, cognitive evaluations were restricted to two tests in the present study to maintain the peak effect of neurostimulation of the CNS. Further evaluations are required to investigate the effect of dual-mode neurostimulation on the rest of the cognitive functions.

Many studies have been conducted on improving athletes' performance using tDCS; however, the role of stimulation of the spinal cord as part of the CNS is not studied as much. A study in this regard investigated the effect of anodal, cathodal and sham tsDCS stimulation on sprint cycling athletes, which demonstrated that cathodal stimulation improved the mean individual strength of the athletes^46^. However, no significant differences were found between sham and anodal stimulation. Although few studies are available on using tsDCS in athletes, a greater number of studies have been performed on healthy and sick individuals using tsDCS, demonstrating the improvement of their performance^2,3^. Another study on healthy subjects showed that cathodal tsDCS could involve more motor units and improve motor function of healthy subjects in comparison with the sham group^3^.

The exact underlying mechanism of tsDCS in improving motor performance is still unknown; however, it is assumed that since tDCS can exert this effect by affecting neurotransmitters^47^, possibly, tsDCS affects the spinal cord similarly. Also, it has been proved that direct current stimulation in the spinal cord leads to immediate polarization of the afferent fiber terminals and modulates the excitatory postsynaptic potentials of afferents to the motor neurons^2^. Inhibition of interneurons of the GABAergic system and thereby increased stimulation of postsynaptic alpha motor neurons is possibly among the reasons for increased stimulation of spinal cord motor units^47^.

Reaction time and accuracy play critical roles in the performance of athletes in many sports fields, playing vital roles in victory or defeat. A slight alteration in the reaction time and prediction skill can remarkably affect the competition's results^27^. The results of novel studies demonstrate that the reaction time of athletes is complex depending on various factors, including age, experience, sports field, and type of stimulus (visual or auditory)^30^. In the current study, the reaction time of experienced athletes (with a minimum of two years of training) was examined. Whether there is a limitation for improvement of the performance of professional athletes is still unknown, which may be clarified by evaluation of world-class and Olympic athletes. The reaction time of the taekwondo practitioners was improved in the present study.

In this study, it was proved that anodal stimulation of the motor cortex and cathodal stimulation of the lumbar spinal cord reduces the reaction time in professional taekwondo practitioners to the designed motor stimuli. These findings were consistent with the other studies in this regard. Seidel et al. demonstrated that anodal stimulation of the motor cortex (M1 region) significantly enhanced the reaction time in the designed test in athletes in comparison with non-athletes^48^.

The participating athletes in this study were soccer players and handball players. They attributed the performance improvement in athletes compared to non-athletes to the higher sensitivity of their motor neurons in the primary motor cortex to direct current stimulation. Another study showed that anodal stimulation of the M1 motor cortex significantly reduced the mean reaction time in the reaction time test^49^. Moreover, they reported that the mentioned effects were attenuated and disappeared 30 min and 60 min after the stimulation, respectively. This observation was attributed to the reduced excitability of the motor cortex and returning to the polarized states of the neurons over the course of time. In the current study, the reaction time of taekwondo practitioners was improved by 4.7%, which can be attributed to the improved excitability of the motor neurons of the primary motor cortex and the spinal cord.

In this study, it was shown that the accuracy of athletes' kicks, used to evaluate their selective attention, was significantly enhanced in the real stimulation compared with the sham stimulation. Since similar observations regarding the reaction time of athletes, it may be concluded that the two variables are totally interrelated and intertwined in athletes. This hypothesis was confirmed in another study by examining the effects of direct current stimulation in basketball practitioners in the implementation of the head-fake technique^50^. They reported that the reaction time of the athlete in this technique was significantly reduced following anodal stimulation of the primary motor cortex (M1) compared to sham and cathodal stimulation, which implies that the accuracy of the athlete is improved in this technique.

However, variable pieces of evidence exist regarding the short-term tDCS effects on exercise performance. Despite the data supporting the positive effects of tDCS on the capacity to acquire skills in sports exercises, some recent studies have raised controversial results. In one study, it was concluded that available evidence does not strongly support the idea that tDCS is an effective tool for improving exercise performance^51^.

A number of reasons have been proposed to explain the controversial results in the literature, including the facts that (1) these studies have used different stimulation parameters and montages, (2) the measured performance differs in different studies, and (3) the results are different in people with different education level. However, the present study showed that dual-mode neurotransmission appeared to be able to effectively improve the performance of taekwondo practitioners.

The potential question arising in the current study is whether dual-mode neurostimulation is considered doping. The controversy of the available evidence raises another question, whether tDCS is eligible as a prohibited procedure under World Anti-Doping Agency rules. It is noteworthy that dual-mode neurostimulation is not considered doping based on the regulation of the World Anti-Doping Agency.

Although this study proved the improved performance of martial sports athletes by dual-mode neurostimulation, this study had some limitations. One of the limitations of the present study was the low sample size of professional taekwondo athletes, not allowing dividing participants into groups of brain stimulation, spinal cord stimulation, and simultaneous brain and spinal cord stimulation groups, which may be done with an increased sample size in future studies. Based on earlier reports in the field of sport, the specialized population limited the sample size of the study. Given the above, the sample size is still too small to be generalizable.

This study paves the way for designing the protocols of CNS stimulation to enhance athletic performance, such as accuracy and reaction time. Given the effect of tsDCS on spinal reflexes, it may possibly enhance motor functions. Considering the positive effects of dual-mode neurotransmission on the athletic performance of professional taekwondo practitioners, it may potentially affect the success of professional athletes in intensive professional competitions. To more accurately evaluate the brain functions in future studies, it is possible to examine the brain map of athletes affected by brain stimulation using various tools, including quantitative electroencephalography (qEEG) or functional magnetic resonance imaging (fMRI). By conducting more studies in this field, the findings of these studies can be provided to related organizations to improve the performances of Olympic and world-class athletes.

## Data Availability

The authors have shared “minimal data set” for the present submission as per the journal’s policy for data availability.
