# Publisher Correction: A dual-mode neurostimulation approach to enhance athletic performance outcome in experienced taekwondo practitioners

**DOI:** 10.1038/s41598-023-28883-8

**Published:** 2023-01-31

**Authors:** Ali‑Mohammad Kamali, Mojtaba Ijadi, Behnam Keshtkarhesamabadi, Milad Kazemiha, Reza Mahmoudi, Amrollah Roozbehi, Mohammad Nami

**Affiliations:** 1 Neuroscience Laboratory, NSL (Brain, Cognition and Behavior), Department of Neuroscience, School of Advanced Medical Sciences and Technologies, Shiraz University of Medical Sciences, Shiraz, Iran; 2 Iranian Neuroscience Society‑Fars Chapter, DANA Brain Health Institute, Shiraz, Iran; 3 Instituto de Investigaciones Científicas Y Servicios de Alta Tecnología (INDICASAT AIP), City of Knowledge, Neuroscience Center, Panama City, Panama; 4 Cellular and Molecular Research Center, Yasuj University of Medical Sciences, Yasuj, Iran; 5 High Performance Brain, Helena Félix Street, No. 7 to 7 D, 1600‑121 Lisbon, Portugal; 6 Harvard Alumni in Healthcare, Harvard University, Boston, MA USA; 7 Brain, Cognition, and Behavior Unit, BrainHub Academy, Dubai, United Arab Emirates

Correction to: *Scientific Reports* 10.1038/s41598-022-26610-3, published online 05 January 2023

The original version of this Article contained errors in the Affiliations.

Mojtaba Ijadi was incorrectly affiliated with ‘Instituto de Investigaciones Científicas Y Servicios de Alta Tecnología (INDICASAT AIP), City of Knowledge, Neuroscience Center, Panama City, Panama.’

The correct affiliation for Mojtaba Ijadi is listed below.

Cellular and Molecular Research Center, Yasuj University of Medical Sciences, Yasuj, Iran.

Behnam Keshtkarhesamabadi was incorrectly affiliated with ‘Cellular and Molecular Research Center, Yasuj University of Medical Sciences, Yasuj, Iran.’

The correct affiliations for Behnam Keshtkarhesamabadi are listed below.

Iranian Neuroscience Society‑Fars Chapter, DANA Brain Health Institute, Shiraz, Iran.

High Performance Brain, Helena Félix Street, No. 7 to 7 D, 1600‑121 Lisbon, Portugal.

Reza Mahmoudi was incorrectly affiliated with ‘High Performance Brain, Helena Félix Street, No. 7 to 7 D, 1600‑121 Lisbon, Portugal.’

The correct affiliation for Reza Mahmoudi is listed below.

Cellular and Molecular Research Center, Yasuj University of Medical Sciences, Yasuj, Iran.

Amrollah Roozbehi was incorrectly affiliated with ‘High Performance Brain, Helena Félix Street, No. 7 to 7 D, 1600‑121 Lisbon, Portugal.’

The correct affiliation for Amrollah Roozbehi is listed below.

Cellular and Molecular Research Center, Yasuj University of Medical Sciences, Yasuj, Iran.

Mohammad Nami was incorrectly affiliated with ‘High Performance Brain, Helena Félix Street, No. 7 to 7 D, 1600‑121 Lisbon, Portugal.’

The correct affiliations for Mohammad Nami are listed below.

Neuroscience Laboratory, NSL (Brain, Cognition and Behavior), Department of Neuroscience, School of Advanced Medical Sciences and Technologies, Shiraz University of Medical Sciences, Shiraz, Iran.

Iranian Neuroscience Society‑Fars Chapter, DANA Brain Health Institute, Shiraz, Iran.

Instituto de Investigaciones Científicas Y Servicios de Alta Tecnología (INDICASAT AIP), City of Knowledge, Neuroscience Center, Panama City, Panama.

Harvard Alumni in Healthcare, Harvard University, Boston, MA, USA.

Brain, Cognition, and Behavior Unit, BrainHub Academy, Dubai, United Arab Emirates.

In addition, affiliation ‘Department of Physiology, School of Medicine, Shiraz University of Medical Sciences, Shiraz, Iran.’ was removed.

As a result, the Affiliations have been renumbered.

The original Article has been corrected.

